# First person – Edward Griffin

**DOI:** 10.1242/dmm.039008

**Published:** 2019-02-15

## Abstract

First Person is a series of interviews with the first authors of a selection of papers published in Disease Models & Mechanisms, helping early-career researchers promote themselves alongside their papers. Edward Griffin is first author on ‘ApoE-associated modulation of neuroprotection from Aβ-mediated neurodegeneration in transgenic *Caenorhabditis elegans*’, published in DMM. Edward conducted the research described in this article while a PhD student in the lab of Guy A. Caldwell and Kim A. Caldwell at The University of Alabama, Tuscaloosa, USA. He is now a postdoctoral fellow in the lab of David Standaert and Ashley Harms at CIRC 516, Birmingham, USA, investigating neurodegeneration, learning, memory and behavior.


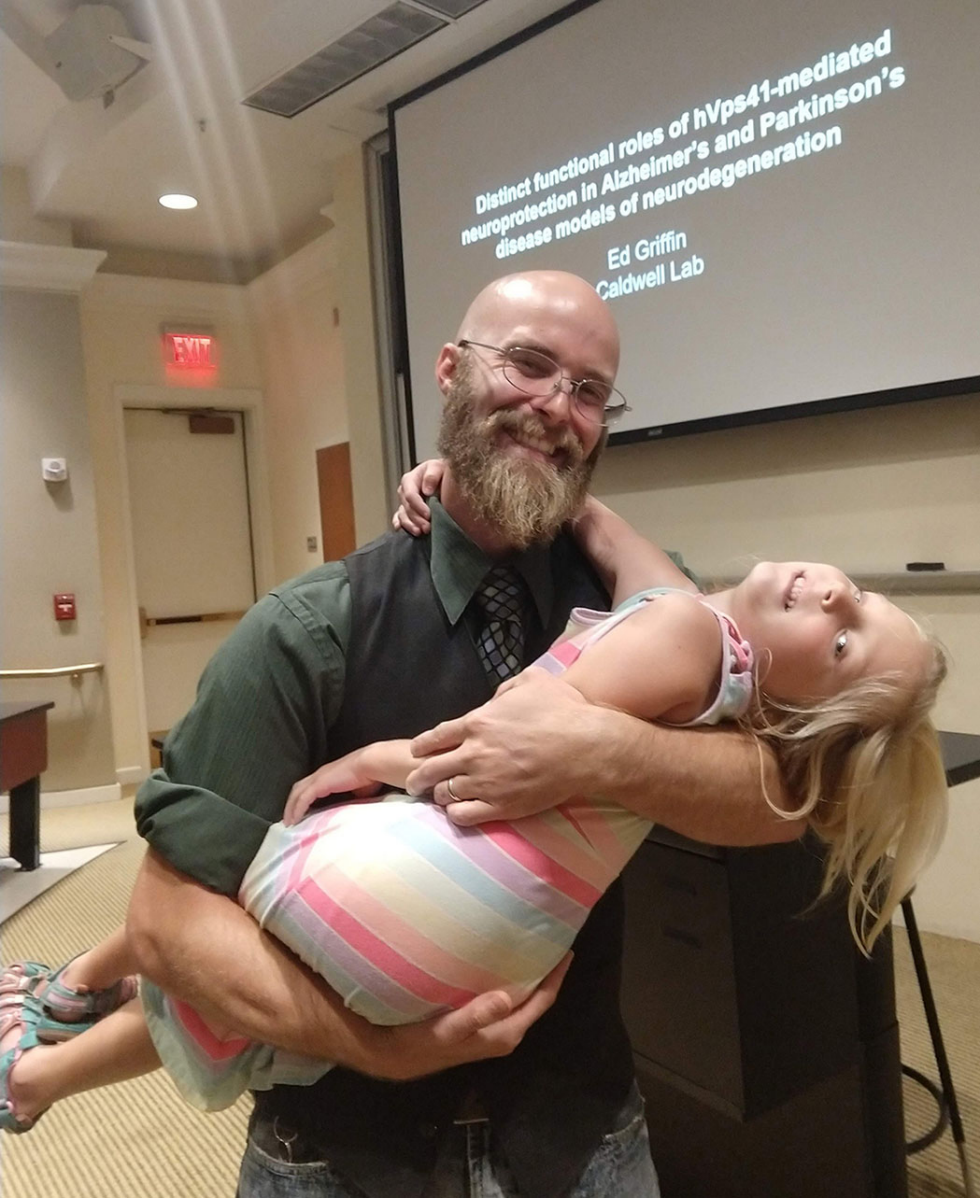


**Edward Griffin**

**How would you explain the main findings of your paper to non-scientific family and friends?**

The cause of Alzheimer's disease is complicated and not well understood, such that the manifestation of the disease often cannot be attributed to just a few causes. Because the disease is also associated with aging, which significantly affects health, it can be challenging to tease apart the variables that contribute to its progression. This having been said, the strongest genetic risk associated with Alzheimer's disease is variation in the gene *APOE*. We sought to make an animal that could model disease progression with ApoE in the neurons of living animals with short lifespans and definitively mapped nervous system. Because ApoE is expressed in many other tissues and involved in metabolic processes between different organ systems, this new organism will be useful in determining how variations in ApoE directly affect neurons in disease.

**What are the potential implications of these results for your field of research?**

It is challenging to examine how this protein affects not just neuronal integrity, but also learning and behavior in a whole animal, without the consequences of other metabolic factors that modify disease progression. With this model, we are able to attribute changes in neurodegeneration and behavior to a handful of cells and what happens within those specific cells. Using this system, we found that calcium homeostasis and ApoE interact in neurodegeneration. Studying this interaction is exciting for us, because it is not well understood how ApoE interacts with calcium to modify disease. Understanding this process better will open up new avenues of understanding and therapeutic targets.

“A surprise that is also a joy is how creative we can be with model systems […] to better understand translatable cellular systems and disease.”

**What are the main advantages and drawbacks of the model system you have used as it relates to the disease you are investigating?**

One advantage is also a drawback, which is that the expression of the ApoE variants is limited to certain cell types in an organism that lacks the endocrine and cholesterol trafficking mechanisms found in mammals. Because we can isolate the effects of this protein from its other functions, that means that its other functions are isolated from the effects we observed in these specific cell types. Additionally, though we can make assumptions how changes in neuronal behavior associated with ApoE might affect learning and cognition, these effects are compounded with the size and sophistication of mammalian brains, which means that translating these neuron-specific effects to the scale of a mammalian brain poses an additional challenge to determining how these effects alter cognition in Alzheimer's disease patients.

**What has surprised you the most while conducting your research?**

I am regularly surprised by how much we do not know, even with those things about which we know the most. Considering the extensive work describing ApoE in cholesterol transport, I did not expect to find such a strong relationship between ApoE and neuronal calcium homeostasis. A surprise that is also a joy is how creative we can be with model systems, whether they be yeast, *Drosophila*, *C. elegans* or cell culture, to better understand translatable cellular systems and disease.

**Figure:**
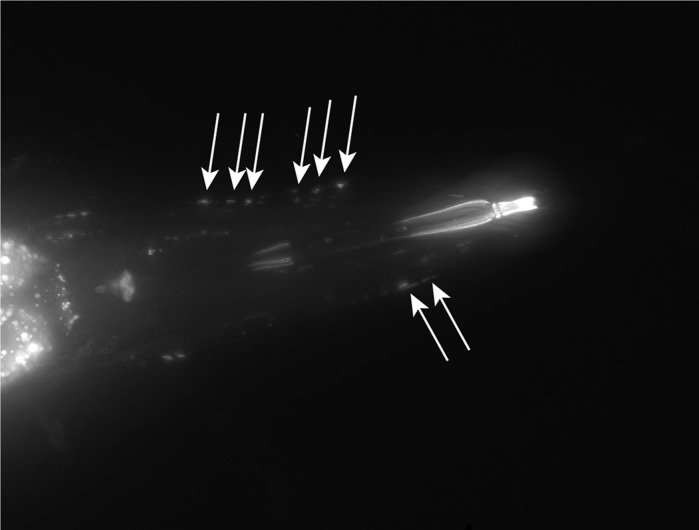
**Aβ plaques (white arrows) illuminated by the fluorescent dye X-37 in *C. elegans* expressing Aβ in the body-wall muscles.**

**Describe what you think is the most significant challenge impacting your research at this time and how will this be addressed over the next 10 years?**

This is a difficult question to answer, because there were challenges 20 years ago we did not expect to be overcome and contribute as significantly as they have. Technologies we think will revolutionize research today may be superseded by other unexpected technologies, much like the Betamax Laserdisc was so quickly succeeded by VHS and DVD. I anticipate that CRISPR technology will indelibly alter the landscape of biological research. A challenge that immediately comes to mind is more primal: that of communication. With the waning of liberal arts in schools, it is becoming more difficult for people to communicate with each other across aisles and societies. For us, that translates into communicating science effectively, whether teaching science or recognizing the limitations of one's model system to cooperate with others whose systems complement their own.

“[…] many would like to start families earlier in their careers, yet feel inhibited by the combination of student debt, income and the demands of graduate school.”

**What changes do you think could improve the professional lives of early-career scientists?**

I may not speak for most, but many would like to start families earlier in their careers, yet feel inhibited by the combination of student debt, income and the demands of graduate school. My graduate institution had a free babysitting program for student parents that was a godsend. Most institutions do not have anything like this. Of course, this is a burden that plagues other careers as well and though it is improving in science, having easier and more affordable access to daycare and family support would help improve the careers of many people who want to pursue science, but feel that it is too difficult to maintain a family early in their career.

**What's next for you?**

I am now a postdoctoral fellow at the University of Alabama at Birmingham. What I do next is uncertain, as I am open to many different opportunities. I think that being open to a variety of possibilities may not only provide flexibility in career goals, but also facilitate happiness, since some goals are not confluent with opportunity.
